# Suprapubic Transvesical Repair of Vesicovaginal Fistula Using a Homemade Laparoscopic Single-Port Device: Experience of 42 Patients

**DOI:** 10.3389/fsurg.2021.744226

**Published:** 2021-11-03

**Authors:** Xinxiang Fan, Xiaoming Ma, Yiming Lai, Zean Li, Jian Huang, Hai Huang

**Affiliations:** ^1^Department of Urology, Sun Yat-sen Memorial Hospital, Sun Yat-sen University, Guangzhou, China; ^2^Guangdong Provincial Key Laboratory of Malignant Tumor Epigenetics and Gene Regulation, Sun Yat-sen Memorial Hospital, Sun Yat-sen University, Guangzhou, China; ^3^Guangdong Provincial Clinical Research Center for Urological Diseases, Guangzhou, China

**Keywords:** transvesical repair, surgical technique, vesicovaginal fistula (VVF), laparoscopic–laparotomy, single-port access

## Abstract

**Aim:** Vesicovaginal fistula (VVF) is the most common urogenital acquired fistula, and has remained a scourge and of public health importance. VVF can be repaired by transvaginal approach, transabdominal approach or transvesical approach, but the optimal management is still debated.

**Methods:** To demonstrate a suprapubic transvesical approach to repair VVFs using a homemade laparoscopic single-port device. A retrospective review of the medical records of 42 consecutive patients who underwent fistula repair for VVF at our center from January 2012 to March 2018 was performed. VVFs were repaired by a suprapubic transvesical approach using a homemade laparoscopic single-port device. Clinical data, perioperative data and outcomes were collected. The primary outcome was VVF successful closure rate, and secondary outcome was perioperative complications.

**Results:** The mean age of the patients was 44.6 (27–58) yr. The mean follow-up time was 65.6 (32–118) mo. The VVFs were successfully closed in 37 (88.1%) patients after the first surgery, and failure was observed in five patients. Initial failures of all the five patients were cured after a second repair. No major complication occurred as defined by Clavien-Dindo class 2 or greater.

**Conclusions:** Suprapubic transvesical approach to repair VVFs using a homemade laparoscopic single-port device is a simple, effective, and feasible approach offering ideal results without major complications.

## Introduction

Urogenital fistula is a global healthcare problem, predominantly associated with obstetric complications in low-resourced countries and iatrogenic injury in well-resourced countries, and vesicovaginal fistula (VVF) is the most common urogenital acquired fistula (1). In developed countries, most VVFs occur after benign gynecological surgery, and hysterectomy for benign vaginal diseases is the most common cause with an incidence ranges from 0.22 to 0.66% (2, 3). In developing countries, most VVFs are associated with labor and delivery (4, 5).

Because the cure rate of conservative treatment is <1%, most VVFs need surgical repair, which can be performed through a transvaginal or a transabdominal approach (6). The transvaginal approach is the most commonly used technique for VVFs repair, especially for primary VVFs, as most gynecologists are more familiar with this approach. In addition, it's also has benefits of less complications, shorter hospital stay and decreased blood loss (7). Nevertheless, if the VVF is located at the vaginal apex, may be hard to access via a transvaginal approach (2). In addition, if the VVF is close to ureteral orifice, the transvaginal approach may increase the risk of ureteral injury. Therefore, there is a growing trend for the management of VVF with a transabdominal approach (open, laparoscopic, or robotic assisted) (1, 2, 5). However, compared with the transvaginal approach, the transabdominal approach has the disadvantages of a longer operative time, more bleeding, longer hospital stay and more major operative complications such as bowel injury (5). Herein, we report a suprapubic transvesical approach to repair VVFs using a homemade laparoscopic single-port device, this novel approach could be easier to access apex VVFs and to avoid ureteral injury compared with the transvaginal approach, and meanwhile could avoid major complications of the transabdominal approach such as bowel injury. The primary outcome of interest was procedure success, defined as absence of leakage at follow-up.

## Patients and Methods

### Study Population

This is a cross-sectional and observational study performed in a high-volume referral center hospital. A total of 42 patients were diagnosed with VVFs after gynecological surgeries between January 2012 and March 2019, who underwent fistula repair by a suprapubic transvesical approach using a homemade single-port device in our department. All surgeries were performed at least 3 months after fistula onset. This study was approved by the Institutional Review Board of our hospital, and informed written consent was obtained from all patients.

### Preoperative Evaluation

A routine preoperative evaluation was performed for all patients, including medical history assessment, physical examination, routine laboratory tests, and methylene blue dye test. Cystovaginoscopy was performed to identify the number, location, size and their relationship of VVFs with ureteral orifices. Computer tomography urography (CTU) was performed to exclude concomitant ureteral injury.

### Surgical Procedure

#### Construction of Homemade Multichannel Single-Port Device

**Figure 1:**
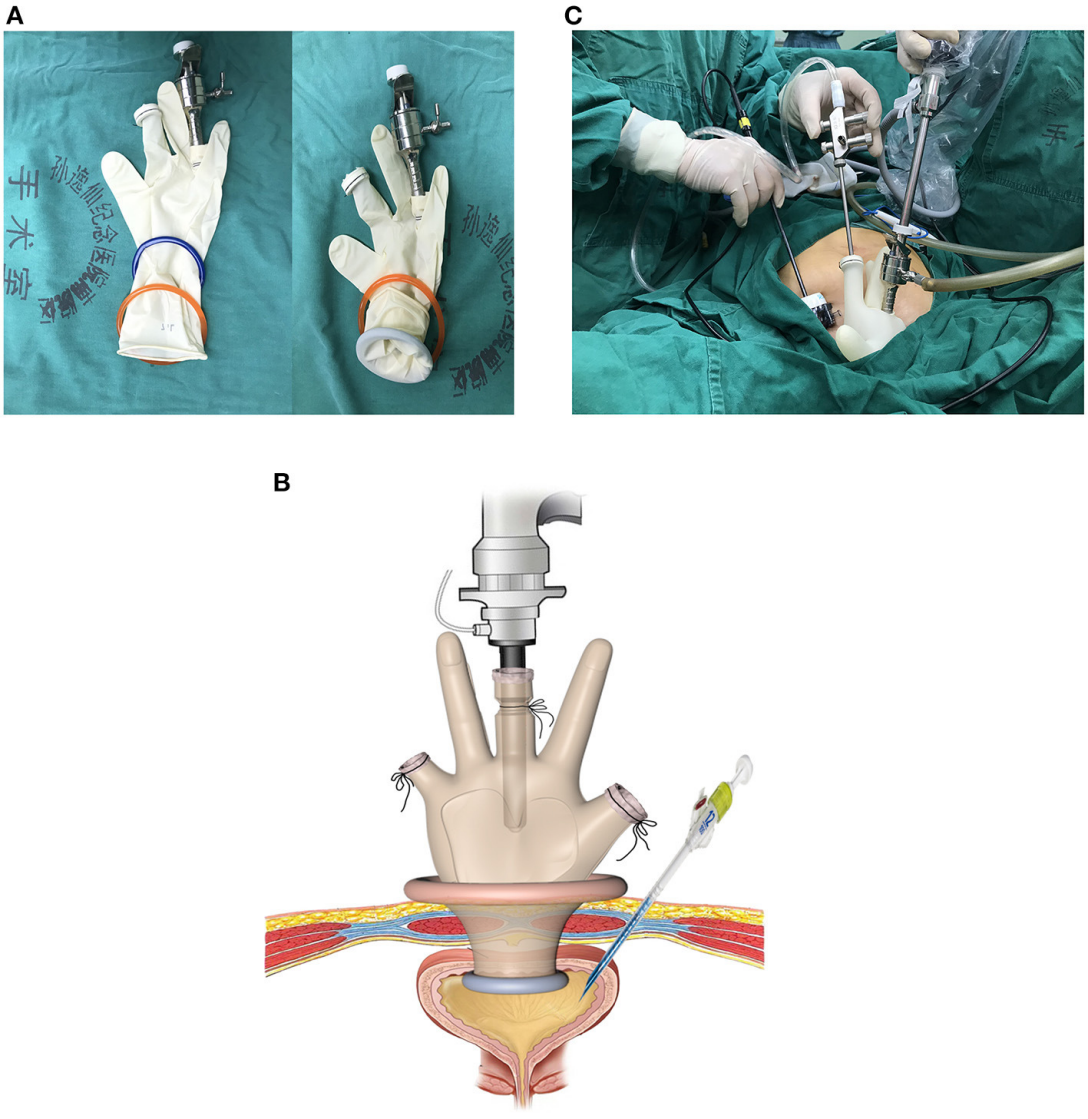
Construction and placement of homemade single-port multi-channel access platform. **(A)** Two stretchable rubber rings and a surgical glove were used to construct the homemade multichannel single-port. **(B,C)** The inner ring was inserted through into the bladder cavity. The outer ring was kept outside the incision. At about 4 cm right lateral to the incision, a 5-mm assistant trocar was placed.

Two stretchable rubber rings, one 4 cm and another 6 cm in diameter, and a surgical glove were used to construct the homemade multichannel single-port (Figure 1A). Two or three fingers of the glove were cut, and one 10 mm metal trocar and another valve compartment of 12 mm trocar were secured with the fingers. The thumb and the little finger were recommended position of the two instrument ports, and sometimes position adjustment might be required according to the position of the fistula. A 15-degree rigid laparoscope (Olympus Surgical, Orangeburg, NY) was inserted through the 10 mm trocar, and the conventional laparoscopic instruments were inserted through another trocar. The 6 cm ring was attached to the cuff of the glove to act as an outer ring. The 4 cm ring was set at 5–6 cm (depending on the thickness of the abdominal wall) from the cuff of the glove and was slipped through the outer ring to form an inner ring. Rubber between the two rings was tightened to prevent CO_2_ leakage (Figure 1B).

#### Establishment of Transvesical Single-Port Access

After general anesthesia, the patients were placed in a Lithotomy position with the feet elevated about 15-degree. A urinary catheter was placed preoperatively, bladder irrigation and vaginal douching was performed. A 4-cm transverse incision was made above the upper edge of pubic symphysis. Next, each layer of the abdominal wall was dissected until the bladder was reached. Then, the bladder was mobilized and incised open longitudinally. The inner ring of the multichannel port was inserted through the incision into the bladder cavity. The outer ring was kept outside the incision. Pneumoperitoneum was obtained by insufflation of CO_2_ to 15 mmHg. The laparoscope was inserted through the access into bladder cavity. At about 4 cm right lateral to the incision, a 5-mm assistant trocar was placed into the bladder under direct laparoscopic vision (Figures 1B,C).

#### Fistula Repair

**Figure 2:**
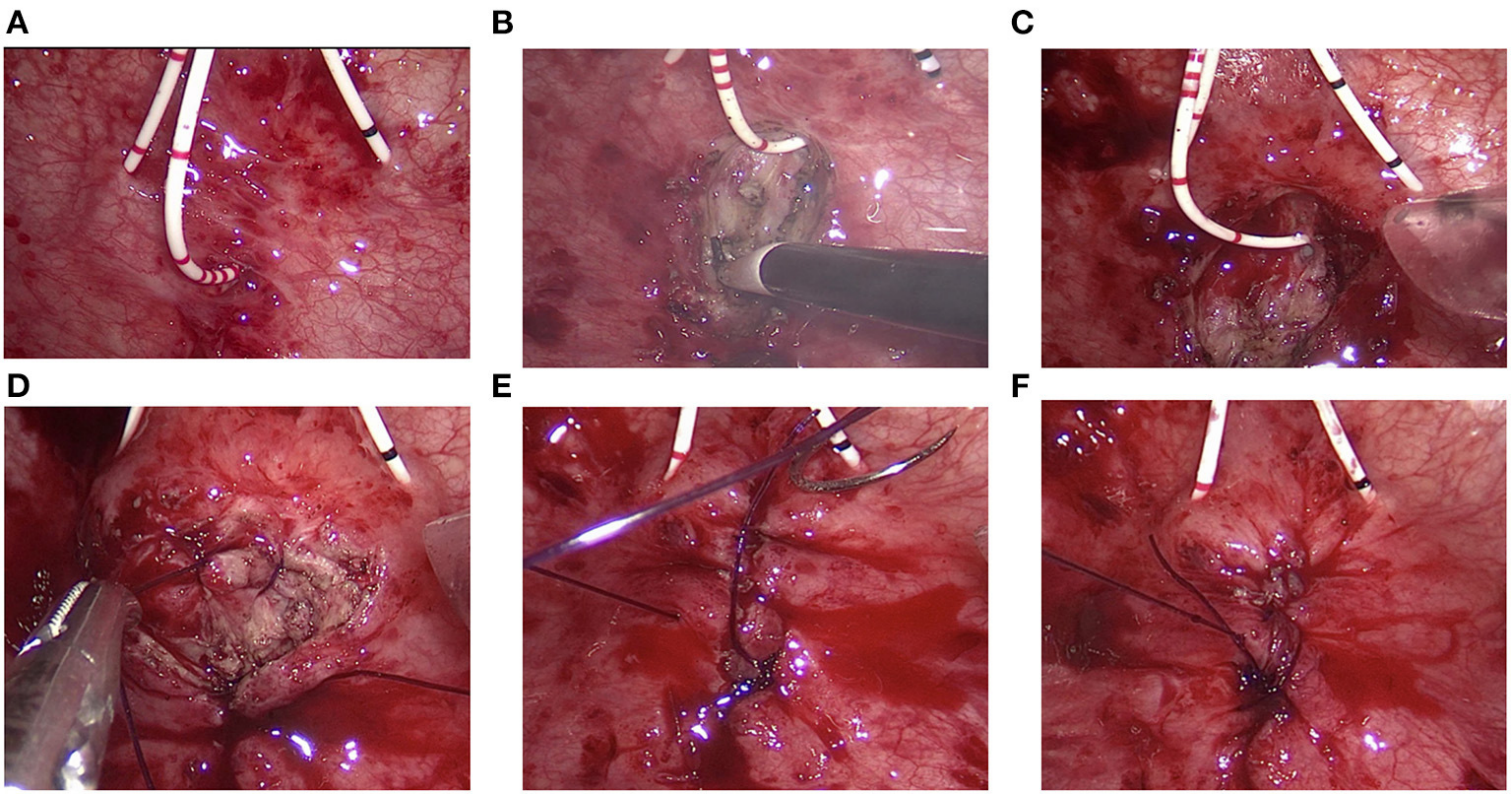
Transvesical repair of vesicovaginal fistula. **(A)** Bilateral ureteral catheters were placed. Another ureteral catheter was inserted into the fistula tract. **(B)** The scar tissue was resected using a coagulation hook. **(C)** The bladder wall was widely mobilized off the vaginal wall. **(D–F)** The vaginal wall, bladder wall, and bladder mucosa were successively closed with 2-0 Monocryl suture.

The bladder cavity was carefully examined to identify ureteral orifices, internal urethral orifice and fistula tract. Ureteral catheters were placed to avoid ureteral injury. Another ureteral catheter was inserted into the fistula tract, which could serve as a guide for resection of the scar tissue of fistula tract (Figure 2A).

The scar tissue at the edge of the fistula orifice was lifted and resected using a coagulation hook (Figure 2B). The bladder wall was widely mobilized off the vaginal wall for a tension-free fistula closure (Figure 2C). The vaginal wall was firstly closed using 2-0 Monocryl suture (Figure 2D). Then, the full layer of the bladder wall was closed with 2-0 Monocryl suture (Figure 2E). Finally, the bladder mucosa is closed with 2-0 Monocryl suture (Figure 2F). In this technique, three layers of closure were made to prevent urine leakage. After that, the ureteral catheter was removed and a urinary catheter was placed. A 12F drainage tube was placed through the 5-mm trocar and removed within 48 h. Single-port access was removed. The bladder wall and abdominal wall were closed successively. Povidone-iodine–soaked sponge is inserted into the vagina and removed the following day.

#### Post-operative Management and Follow-Up

Patients are discharged from the hospital within 5–7 days, antibiotics are prescribed for 14 days, antimuscarinics such as Solinasin or Tolterodine are given to the patients for 4 weeks, and Foley catheter drainage is maintained for 2 weeks. All patients underwent a regularly follow-up in accordance with relevant guidelines and regulations. Symptom assessment and physical examination was performed at every visit. Cystovaginoscopy was performed if patient still complained of urine leakage. Complications of the procedure were assessed according to the patients' medical records and form the direct description of patients during follow-up. The complications were classified according to the Clavien-Dindo classification (8, 9).

## Results

The characteristics of patients and fistulas are shown in Table 1. A total of 42 patients diagnosed with VVFs underwent fistula repair in our department, with a mean age of 44.6 (range: 27–58) yr. Among these 42 patients, 33 were primary VVF patients (no prior treatment), whereas nine were recurrent VVF patients (one to two attempts of repair by transvaginal approach at gynecology department). The mean fistula size was 0.9 ± 0.5 cm. The most common location of VVFs was the supratrigonal posterior bladder wall (29 cases, 69.0%). Among the patients, 37 had a single fistula, and five had two or more fistulas.

**Table 1:** Demographic and clinical characteristics of 42 patients.

**Factor**	**Value**
Total number of patients	42
Age (years), mean	44.6 (27–58)
Fistula size (mm), mean ± SD	0.9 ± 0.5
Fistula number	
Single	37
Multiple	5
Fistula history	
Primary fistula	33
Secondary fistula	9
Location, n (%)	
Supratrigonal	29 (69.0%)
Trigonal	13 (31.0%)
Etiology of fistula, n (%)	
Cervical cancer	21 (50.0%)
Myoma	15 (35.7%)
Adenomyosis	3 (7.1%)
Endometrial cancer	1 (2.4%)
Ureterostenosis	1 (2.4%)
Cesarean	1 (2.4%)
Surgeries leading to fistula	
Panhysterectomy	26 (61.9%)
Radical hysterectomy	15 (35.7%)
Ureteral reimplantation	1 (2.4%)

The mean operative time was 41.3 ± 12.9 min. Suprapubic transvesical repair were successfully completed in 41 patients. One patient was converted to transabdominal approach due to two large fistulas, as the pneumoperitoneum could not be sustained. The procedure was defined as success if the patient had no urine leakage during the follow-up period. The mean follow-up time was 65.6 (32–118) mo. The VVFs were successfully closed in 37 (88.1%) patients after the first surgery, and failure was observed in five patients. Reasons of failure including three patients had a pelvic radiation therapy history, and two patients had a large fistula (>2 cm). Initial failures of all the five patients were cured after a second repair. No major complication occurred as defined by Clavien-Dindo class 2 or greater. There was also no bowel, ureteral, or nerve injury. Minor complications according to Clavien-Dindo class 1 were shown in Table 2. Common minor complications included bladder spasm (3, 7.1%) and urinary tract infections (5, 11.9%). Incision infection occurred in an obese patient (BMI:28.6) due to fat liquefaction.

**Table 2:** Operative and post-operative data.

**Parameters**	**Value**
Mean operative time (min), mean ± SD	41.3 ± 12.9
Mean Length of hospital stay (d), mean ± SD	5.4 ± 2.2
Success rate, no. (%)	
First repair	37/42 (88.1%)
Second repair	5/5 (100%)
Complications	
Major complications	0
Minor complications	11 (26.2%)
Symptomatic bladder spasms (n, %)	3 (7.1%)
Urinary tract infections (n, %)	5 (11.9%)
Hematuria	2 (4.8%)
Infection in incision area	1 (2.4%)

## Discussions

VVF is the most frequently acquired fistula, and has remained a scourge and of public health importance, not just for the attendant medical and physical disabilities, but also for the inherent social, emotional and psychological strain, and stress on the victims (5).

Any surgical procedure in the pelvis can lead to fistulae formation. A patient might be suspected to have a VVF when she has urine leak 1–2 week postoperatively. VVF is often because of tissue necrosis captured in surgery or due to cautery. It must be emphasized that VVFs are not necessarily a consequence of inadvertent organ injury or surgical misadventure. Tissue necrosis may occur because of extensive dissection or haematoma formation with fistulae forming weeks later. VVFs that result following radiation therapy may present many months to years later and are thought to occur due to chronic small vessel inflammatory changes leading to tissue ischaemia (1). Diagnosis can be established by filling the bladder with methylene blue, inserting a tampon in the vagina and asking the patient to ambulate (2). Cystoscopy and vaginoscopy have prime importance to check the location, size, number of fistulas, and evaluate the surrounding tissue, which helps in the future management plan. Surgery should be postponed if there are signs of acute inflammation, edema, necrosis, or other pathology of the vagina or bladder. If there is a suspicion of concomitant ureteral injury, CTU or MRU is recommended. In long-standing patients of VVFs, or repeated recurrent VVFs, bladder capacity can be reduced, and this might change the management plans.

VVFs can be best managed following principles, such as adequate exposure, identification of structures, wide mobilization, tension-free closure, good hemostasis and uninterrupted bladder drainage (5). The choice of surgical procedures depends on the surgeon's experience, location and size of the fistula, and patients' preferences. Most gynecologists prefer the vaginal approach, which has the benefits of minimal blood loss, shorter hospital stay and relatively less postoperative morbidity, a higher success rate, and has little affect to secondary surgery if the first surgery failed comparable with the abdominal approach (10–12). However, the vaginal approach also has some limitations, as it is difficult to perform when there is vaginal stenosis, improper exposure or synchronous ureteric involvement. In addition, the risk of ureter injury increase if the fistula is close to the ureteral orifice. Many urologists choose the transabdominal or transvesicle approach due to a lack of familiarity with the vaginal repair.

Laparoscopic transvesicle VVFs repair was first reported by Wong et al. in 2006 (13). Roslan et al. (14) reported a single case of transvesicle LESS VVFs repair in 2012, which demonstrate the feasibility of LESS surgery. Based on the experience of many LESS surgeries in urology, we carried out transvesicle LESS VVFs repair with a homemade single-port in 42 patients from January 2012. To our knowledge, this is the largest reported series of transvesicle LESS VVFs repair. In this study, VVFs were successfully closed in 88.1% cases after the first repair and all five cases that initially failed were cured with a second repair. Based on our experience, it is easier to repair fistulas in these failed cases compared with first-time cases, because the failed cases have single and smaller fistula after the initial repair.

The important surgical principles are included in the article to demonstrate the successful closure of a VVF and the good functional results. During this approach, there is no need to interfere the abdominal cavity, thus avoid the risk of bowel injury when isolating the adhesive abdominal viscera. Through the establishment of pneumoperitoneum directly into the bladder cavity, and dissection of the scar tissue of the fistula, isolating the vaginal wall and the bladder wall, and then suture them separately. This procedure greatly simplifies the previous intraperitoneal surgery. Compared with the transvaginal approach, we have a clear vision of the relationship between the fistula orifice and the ureteral orifice, avoiding the possibility of damage to the ureter. Therefore, the transvesicle approach is a simple and feasible approach to most VVFs.

This study has the following limitations. First, this is a retrospective study and the comparison of the LESS approach with the conventional suprapubic transvesical approach or transabdominal approach is still needed in the future. Second, the laparoscopic single-port surgery is more challenging for surgeons. It is difficult to use the conventional laparoscopic instrument in single-port especially suture. The angle of the needle holder and the forceps are quite important. It is not suitable for larger fistulas, concomitant ureteral injuries, or severe damage infection and adhesion of fistula's surrounding tissue. Third, our device lacks standardization in particular in the use of the two rings, and our next goal is to make commercial standardized device.

## Conclusions

Our study demonstrates that suprapubic transvesical approach to repair VVFs using a homemade laparoscopic single-port device is a simple, effective, and feasible approach offering ideal results without major complications.

## Data Availability Statement

The original contributions presented in the study are included in the article/supplementary material, further inquiries can be directed to the corresponding authors.

## Ethics Statement

The studies involving human participants were reviewed and approved by Sun Yat-sen Memorial Hospital. The patients/participants provided their written informed consent to participate in this study.

## Author Contributions

XF and XM: conceptualization, formal analysis, investigation, writing—original draft, writing—review and editing, and visualization. ZL: formal analysis, investigation, and visualization. YL: investigation and formal analysis. JH and HH: conceptualization, investigation, data curation, writing—review and editing, supervision, funding acquisition, and project administration. All authors contributed to the article and approved the submitted version.

## Funding

This study was funded by the National Natural Science Foundation of China (82002682), the Natural Science Foundation of Guangdong (2015A030310090, 2016A030313291), and the Guangdong Provincial Clinical Research Center for Urological Diseases (2020B1111170006).

## Conflict of Interest

The authors declare that the research was conducted in the absence of any commercial or financial relationships that could be construed as a potential conflict of interest.

## Publisher's Note

All claims expressed in this article are solely those of the authors and do not necessarily represent those of their affiliated organizations, or those of the publisher, the editors and the reviewers. Any product that may be evaluated in this article, or claim that may be made by its manufacturer, is not guaranteed or endorsed by the publisher.
